# Patch-Seq Protocol to Analyze the Electrophysiology, Morphology and Transcriptome of Whole Single Neurons Derived From Human Pluripotent Stem Cells

**DOI:** 10.3389/fnmol.2018.00261

**Published:** 2018-08-10

**Authors:** Mark van den Hurk, Jennifer A. Erwin, Gene W. Yeo, Fred H. Gage, Cedric Bardy

**Affiliations:** ^1^Laboratory for Human Neurophysiology and Genetics, South Australian Health and Medical Research Institute (SAHMRI) Mind and Brain, Adelaide, SA, Australia; ^2^The Lieber Institute for Brain Development, Baltimore, MD, United States; ^3^Department of Neurology, School of Medicine, Johns Hopkins University, Baltimore, MD, United States; ^4^Department of Cellular and Molecular Medicine, University of California, San Diego, La Jolla, CA, United States; ^5^Department of Physiology, Yong Loo Lin School of Medicine, National University of Singapore, Singapore, Singapore; ^6^Laboratory of Genetics, Salk Institute for Biological Studies, Sanford Consortium for Regenerative Medicine, La Jolla, CA, United States; ^7^Flinders University College of Medicine and Public Health, Adelaide, SA, Australia

**Keywords:** patch-seq, single-cell RNA-seq, induced pluripotent stem cell (iPSC), human neuron transcriptome, neuronal diversity, cellular phenotyping, patch clamping, electrophysiology

## Abstract

The human brain is composed of a complex assembly of about 171 billion heterogeneous cellular units (86 billion neurons and 85 billion non-neuronal glia cells). A comprehensive description of brain cells is necessary to understand the nervous system in health and disease. Recently, advances in genomics have permitted the accurate analysis of the full transcriptome of single cells (scRNA-seq). We have built upon such technical progress to combine scRNA-seq with patch-clamping electrophysiological recording and morphological analysis of single human neurons *in vitro*. This new powerful method, referred to as Patch-seq, enables a thorough, multimodal profiling of neurons and permits us to expose the links between functional properties, morphology, and gene expression. Here, we present a detailed Patch-seq protocol for isolating single neurons from *in vitro* neuronal cultures. We have validated the Patch-seq whole-transcriptome profiling method with human neurons generated from embryonic and induced pluripotent stem cells (ESCs/iPSCs) derived from healthy subjects, but the procedure may be applied to any kind of cell type *in vitro*. Patch-seq may be used on neurons *in vitro* to profile cell types and states in depth to unravel the human molecular basis of neuronal diversity and investigate the cellular mechanisms underlying brain disorders.

## Introduction

Neurons represent the basic functional units of the nervous system and are unique in many aspects, including their morphological and physiological properties (Kawaguchi, 1993; Pennartz et al., 1998; Faber et al., 2001; Hamam et al., 2002). A comprehensive, transcriptome-based deconvolution of complex tissue into cellular subtypes is required to better understand cellular mechanisms and behavior in healthy, developmental, and disease states. Several approaches have been developed to profile individual neurons at the physiological and molecular level. In the past, the combination of patch-clamp with single-cell reverse transcription PCR (Eberwine et al., 1992; Lambolez et al., 1992; Cauli et al., 1997; Sucher et al., 2000; Toledo-Rodriguez et al., 2004; Weng et al., 2010; Citri et al., 2011; Belinsky et al., 2014) or microarray (Subkhankulova et al., 2010) techniques has enabled the correlation of cellular function with gene expression patterns. These techniques, however, only provide a snapshot of the expression of a limited number of pre-specified genes, precluding an unbiased discovery of novel transcripts and splice variants. With the recent advances in whole-transcriptome amplification and next-generation sequencing methods, the profiling of single-cell transcriptomes by RNA sequencing (i.e., scRNA-seq) has become a promising and prevailing approach to define and disentangle in greater detail the heterogeneity of cell types (Eberwine et al., 2014).

To identify molecular features associated with specific neuronal functions and phenotypes, we and others have combined scRNA-seq profiling with electrophysiological and morphological characterization of individual neurons in a method referred to as Patch-seq (Bardy et al., 2016; Cadwell et al., 2016; Chen et al., 2016; Foldy et al., 2016; Fuzik et al., 2016; Cadwell et al., 2017). Neurological or psychiatric disease-specific abnormalities generally manifest only in particular (sub)types of neurons or at specific maturational stages, and Patch-seq provides the resolution to identify such unique cell types (Stahlberg et al., 2011; Eberwine et al., 2014). Unlike more automated single-cell capture techniques (e.g., microfluidics systems or droplet-based sequencing approaches), Patch-seq enables the transcriptome data to be linked to quantifiable neurophysiological phenotypes (electrophysiology and neuro-morphology).

Here, we describe our Patch-seq protocol, which we previously validated in a thorough analysis of human iPSC-derived neurons from healthy subjects (Bardy et al., 2016). In contrast to other published approaches (Cadwell et al., 2016; Fuzik et al., 2016; Cadwell et al., 2017; **Table 1**), our protocol includes the analysis of cytosolic RNA as well as distant RNA in the dendrites and axon. Microscopic examination demonstrates that the entire neuronal soma and the neurites (≥150 μm distance away from the cell body) are collected with our approach. By including transcripts from adjacent neurites, this method presumably provides a more accurate representation of the complete transcriptional program of the cell (Cajigas et al., 2012), making it potentially valuable for studying neurobiological mechanisms or disorders relying on distal synaptic/dendritic mRNA trafficking (Bagni and Greenough, 2005; Bassell and Warren, 2008). The method is particularly well suited for the collection of single neurons cultured in a petri dish or on a glass coverslip, such as *in vitro* stem cell-derived neurons or primary neurons.

**Table 1:** Comparison of Patch-seq methods for multimodal profiling of single neurons.

	Patch-seq Bardy et al. (2016)	Patch-seq Cadwell et al. (2016, 2017)	Patch-seq Fuzik et al. (2016)
Patch-seq-analyzed cell type(s)	*In vitro* ESC/iPSC-derived human midbrain-like neurons	*Ex vivo* and *in vivo* mouse neocortex interneurons	*Ex vivo* mouse neocortex pyramidal cells and interneurons
Precautions against RNase	✓	✓	?
Transcriptome sampling method	Entire neuron isolation including dendrites and axon	Aspiration of cell soma contents	Aspiration of cell soma contents
Analysis of transcripts from distal neurites	✓	χ	χ
Addition of RNase inhibitor to internal solution (to reduce RNase activity)	χ	✓	χ
Addition of EGTA to internal solution (to chelate divalent cations that are cofactors for RNase)	✓	✓	✓
Single-cell RNA reverse-transcribed and amplified immediately (<4 h) after collection	✓	?	χ
scRNA-seq protocol	Smart-seq (SMARTer)	Smart-seq2	STRT-C1

To confirm successful collection of the entire neuron and to ensure that only one cell is captured in each sample, our neuronal isolation method with a micropipette relies on visual microscopic confirmation. Following collection of a single neuron, the sample is immediately processed for cDNA synthesis and amplification, thereby avoiding any possible degradation of the picogram amounts of mRNA and optimizing the accuracy of acquiring a snapshot of the single-cell transcriptome. Procedural effects on cell molecular profiles are minimized as the neurons remain functional in their spatial context and environmental niche until the final collection step, which can be completed in under a minute. In contrast, microfluidic devices used to collect single cells for scRNA-seq are more disruptive to the cells (i.e., neurons dissociated with cut dendrites) and ultimately less accurate than the Patch-seq protocol described here. However, automated microfluidic systems can be a valuable complement to Patch-seq by providing a less labor-intensive and higher throughput analysis of single-cell transcriptomic profiles.

A thorough quality control (QC) analysis of Patch-seq is performed on each reverse-transcribed and PCR-amplified sample prior to library preparation and deep transcriptome sequencing to identify and filter out spurious captures and contaminated cell samples. The QC pipeline described here is based on (i) expression profiling of common housekeeping genes and differentially abundant exogenous reference transcripts (ERCC spike-in controls), (ii) fluorometric quantitation of cDNA yield (Qubit), and (iii) qualitative analysis of cDNA fragment profiles (Agilent Bioanalyzer).

### Current and Future Applications of Patch-seq

Patch-seq analysis allows correlation between gene expression profiles, physiological function, and morphology of single cells. So far, Patch-seq has been successfully applied to human neuronal cultures *in vitro* (Bardy et al., 2016) and *ex vivo* rodent brain slices (Cadwell et al., 2016; Fuzik et al., 2016). In the future, Patch-seq analysis might also be used to profile live human neurons obtained from patients via surgical biopsy. The application of Patch-seq for multimodal classification of neuronal types in the mouse brain (Cadwell et al., 2016; Fuzik et al., 2016) is very likely to complement global efforts in classifying all the cell types in the human brain (Ecker et al., 2017). To further elucidate neuronal circuitry structure and function, Patch-seq performed on rodent brain slices may be combined with optical tools or synapse-specific trans-neuronal tracing methods (Ginger et al., 2013; Kim et al., 2016) to interrogate the transcriptome of neurons receiving inputs from, or projecting to, specific areas in the brain.

Patch-seq has also been used to study human neurodevelopmental mechanisms with iPSCs *in vitro* (Bardy et al., 2016) and, in the future, is very likely to serve investigation aiming to decipher a wide range of fundamental cellular mechanisms in both health and disease. Intra- and inter-donor sample variability in RNA-seq experiments is a serious challenge and we believe that Patch-seq can help addressing this by reducing the heterogeneity of cell types in transcriptomic analysis (Hoffman et al., 2017). Finally, Patch-seq may be extended to non-neuronal cells which are electrophysiologically interesting, such as iPSC-derived cardiomyocytes, which can also be cultured on coverslips (Ma et al., 2011; Davis et al., 2012).

### Advantages of Patch-seq Analysis of Human Pluripotent Stem Cell-Derived Neurons *in vitro*

The approach we developed to thoroughly analyze the phenotypes of human ESC/iPSC-derived neurons *in vitro* using Patch-seq is innovative and sets a new standard for neurobiological analysis in the following ways:

• Enabling a precise identification and multimodal characterization of cellular subtypes. Patch-seq provides a complete phenotypic analysis, including electrophysiology, morphology, and transcriptomic profiles of single neurons. Cell biological functions are intertwined and depend on each other, and Patch-seq multimodal profiling of single cells will allow thorough analysis of inter-dependent neurobiological processes.
• Resolving bias from bulk analysis of cells/tissues. *En masse* pooling and analysis of cells obscures subpopulations and does not accurately reflect the biological changes that happen at the single-cell level (Toriello et al., 2008; Wills et al., 2013; Patel et al., 2014; Sandberg, 2014). Combining electrophysiological and morphological analysis of single neurons with single-cell expression profiling provides the resolution required for identifying rare or clinically important cell types and the aberrant molecular mechanisms associated with them (Stahlberg et al., 2011; Eberwine et al., 2014).
• Eliminating bias resulting from variability in tissue culture conditions. A major hurdle to overcome in using human iPSC disease models is inherent tissue culture variability. In particular, human neuronal models, which require extended time for maturation *in vitro* (>1 month), are inherently variable and are often characterized by a considerably heterogeneous proportion of functionally mature neurons (Bardy et al., 2016). In combination with rigorous control and optimization of overall tissue culture conditions, a molecular analysis of neurons pre-characterized with patch clamping accelerates the discovery of new neuron type-specific biomarkers, which can be used to reduce immanent phenotypic variability and streamline more accurate investigations in higher throughput fashion.

## Materials and Equipment

### Reagents

#### Coverslip Preparation and Coating Reagents

• Nitric acid (Sigma-Aldrich, 4388073)
   ◦ Caution: Nitric acid is extremely corrosive; handle with care and use appropriate personal protective equipment.

• Hydrochloric acid (Sigma-Aldrich, 258148)
   ◦ Caution: Hydrochloric acid is extremely corrosive; handle with care and use appropriate personal protective equipment.

• Ethanol (Chem-Supply, EL043-2.5L-P)
• Poly-L-ornithine hydrobromide (Sigma-Aldrich, P3655)
• Natural mouse laminin (Thermo Fisher Scientific, 23017015)
• D-PBS without Ca++ and Mg++ (STEMCELL Technologies, 052014)

#### Cell Culture Reagents

• DMEM/F-12+GlutaMAX medium (Thermo Fisher Scientific, 10565018)
• BrainPhys Neuronal Medium (STEMCELL Technologies, 05790)
• N-2 Supplement (100×) (Thermo Fisher Scientific, 17502048) or N2 Supplement-A (100×) (STEMCELL Technologies, 07152)
• B-27 Supplement (50×) (Thermo Fisher Scientific, 17504044) or NeuroCult SM1 Neuronal Supplement (50×) (STEMCELL Technologies, 05711)
• FGF8 (Peprotech, 100-25)
• SHH (R&D Systems, 1314SH)
• BDNF (Peprotech, 450-02)
• GDNF (Peprotech, 450-10)
• L-Ascorbic acid (Sigma-Aldrich, A4403)
• Dibutyryl cyclic AMP (Sigma-Aldrich, D0627)
• Natural mouse laminin (Thermo Fisher Scientific, 23017015)
• Accutase (STEMCELL Technologies, 07920)
• Matrigel hESC-qualified matrix (Corning, 354277)

#### Patch Clamping Internal Solution Reagents

• Potassium D-gluconate (Sigma-Aldrich, G4500)
• Potassium chloride (Sigma-Aldrich, 60128)
• Sodium chloride (Sigma-Aldrich, S6191)
• HEPES sodium salt (Sigma-Aldrich, H3784)
• EGTA (Sigma-Aldrich, E3889)
• Guanosine 5′-triphosphate sodium salt hydrate (Na-GTP) (Sigma-Aldrich, G8877)
• Adenosine 5′-triphosphate magnesium salt hydrate (Mg-ATP) (Sigma-Aldrich, A9187)
• Dextrose (Sigma-Aldrich, G7021)
• Biocytin (Sigma-Aldrich, B4261)
• Rhodamine B isothiocyanate–dextran (Sigma-Aldrich, R8881)
• DNase/RNase-free distilled water (Thermo Fisher Scientific, 10977015)
• Potassium hydroxide solution, 8.0 M (Sigma-Aldrich, P4494)
• D-Gluconic acid solution, 49–53 wt.% in water (Sigma-Aldrich, G1951)

#### RNA Isolation, cDNA Synthesis, and Library Preparation Reagents

• RNase AWAY Decontamination Reagent (Thermo Fisher Scientific, 10328011)
• SMARTer Ultra Low Input RNA for Illumina Sequencing – High Volume kit (Clontech, 634828)
• ERCC RNA Spike-In Mix (Thermo Fisher Scientific, 4456740)
• Nextera XT DNA Library Preparation Kit (Illumina, FC-131-1096)
• Nextera XT Index Kit (Illumina, FC-131-1001)
• Agencourt AMPure XP magnetic beads (Beckman Coulter, A63881)
• SYBR Gold Nucleic Acid Gel Stain (Thermo Fisher Scientific, S11494)
• Wizard SV Gel and PCR Clean-Up System (Promega, A9282)
• Ethanol, molecular biology grade (Sigma-Aldrich, E7023)
• DNase-/RNase-free distilled water (Thermo Fisher Scientific, 10977015)

#### cDNA Quality Control Reagents

• Qubit dsDNA High-Sensitivity Assay Kit (Thermo Fisher Scientific, Q32854)
• Qubit Assay Tubes (Thermo Fisher Scientific, Q32856)
• Human GAPD (GAPDH) Endogenous Control, FAM/MGB probe, non-primer limited (Thermo Fisher Scientific, 4333764F)
• Human ACTB (Beta Actin) Endogenous Control, FAM/MGB probe, non-primer limited (Thermo Fisher Scientific, 4333762F)
• TaqMan Gene Expression Master Mix (Thermo Fisher Scientific, 4369510)
• High Sensitivity (HS) DNA Kit – Bioanalyzer Chips & Reagents (Agilent, 5067-4626)
• KAPA Library Quantification Kit for Illumina Platforms (KAPA Biosystems, KK4835)

### Equipment and Supplies

#### Cell Culture Equipment

• CO_2_ incubator (Thermo Fisher Scientific, HERAcell VIOS 160i)
• Biological safety cabinet
• Sonicator (Soniclean, 250T)
• Countess II FL automated cell counter (Thermo Fisher Scientific, AMQAF1000) or hemacytometer (Sigma-Aldrich, Bright-Line)
• Centrifuge (Sigma, 3-16KL)
• Inverted phase contrast microscope with fluorescence (Olympus, IX73)

#### Patch Clamping and Imaging Equipment

• Flaming/Brown type micropipette puller (Sutter Instrument, P-1000)
• Temperature controller with in-line Peltier heater (Scientifica, SM-4600)
• Moving XY stage platform with joystick control (Scientifica)
• Motorized micromanipulators (Scientifica, PatchStar/MicroStar)
• MultiClamp 700B microelectrode amplifier (Molecular Devices)
• Digidata 1550B low noise data acquisition system (Digidata, 1550B4)
• Fixed-stage upright microscope with fluorescence, infrared and DIC (Olympus, BX51WI)
• Fluorescence microscopy illumination system (CoolLED, PE-4000-L-SYS)
• Scientific CMOS camera (Photometrics, Prime 4.2 sCMOS)

#### Molecular Biology and General Equipment

• RNA clean hood
• Thermal cycler for PCR (Bio-Rad, T100)
• Real-time PCR detection system (Thermo Fisher Scientific, ABI PRISM 7900HT)
• PCR sample cooler (Eppendorf, 3881000015)
• DynaMag-PCR magnet for 0.2-ml PCR tubes (Thermo Fisher Scientific, 492025)
• DynaMag-2 magnet for microcentrifuge tubes (Thermo Fisher Scientific, 12321D)
• Qubit 2.0 fluorometer (Thermo Fisher Scientific, Q32866)
• 2100 Bioanalyzer Instrument (Agilent, G2939BA)
• HiSeq 2500 next-generation sequencing instrument (Illumina, SY-401-2501)
• Microcentrifuge (Thermo Fisher Scientific, mySPIN 6)
• Vortex (Thermo Fisher Scientific, LP Vortex mixer)
• SpeedVac Concentrator
• Milli-Q lab water purification system
• Pipettors (Gilson, Pipetman)
• Micro Jeweler forceps with delicate tips (INKA Surgical Instruments, 12453.45)
• 1-l heavy duty borosilicate glass beaker (Corning, 1003-1L)
• Low-profile clear glass jars with closure for coverslip storage (Thermo Fisher Scientific, 120-0250)
• Ice bucket

#### Consumables

• Nonstick, RNase-free 1.5-ml microfuge tubes (Thermo Fisher Scientific, AM12450)
• Sterile filter pipette tips 10, 30, 100, 200, and 1000 μl (Gilson, DIAMOND)
• Sterile serological pipettes 2, 5, 10, and 25 ml (Corning, 4486-4489)
• Sterile aspirating pipettes 2 ml (Corning, 9186)
• Thin-walled 0.2-ml PCR tubes (Bio-Rad, TFI-0201)
• Borosilicate capillary glass with filament, o.d. 1.50 mm, i.d. 0.86 mm, length 100 mm (Sutter Instrument, BF150-86-10)
• Parafilm (Sigma-Aldrich, P7793)
• 96-well PCR reaction plates (Thermo Fisher Scientific, MicroAmp)
• 48-well tissue culture plates, sterile, tissue culture-treated (Corning, 3548)
• 24-well tissue culture plates, sterile, tissue culture-treated (Corning, 3524)
• Conical tubes 15 ml (Corning, 430828)
• Conical tubes 50 ml (Corning, 430828)
• Coverslips for 24-well plates, 12 mm diameter, No. 1 thickness (ProScitech, G401-12)
• Coverslips for 48-well plates, 8 mm diameter, No. 1 thickness (ProScitech, G401-08)
• Sterile disposable reagent reservoirs (Corning, 4870)
• Delicate task kimwipes (KimTech Science by Kimberly Clark, 34133)
• Saran wrap

### Reagent Setup

• Neural Progenitor Cell Medium (NPM): DMEM/F12+GlutaMAX medium supplemented with 1× N-2 (or N2 Supplement-A), 1× B-27 (or NeuroCult SM1), 100 ng/ml FGF8, 200 ng/ml SHH, and 1 μg/ml laminin
• Neural Differentiation Medium (NDM): BrainPhys Neuronal Medium supplemented with 1× N-2 (or N2 Supplement-A), 1× B-27 (or NeuroCult SM1), 20 ng/ml BDNF, 20 ng/ml GDNF, 200 nM ascorbic acid, 1 mM dibutyryl cyclic AMP, and 1 μg/ml laminin
• Patch clamping internal solution: 130 mM K-gluconate, 6 mM KCl, 4 mM NaCl, 10 mM Na-HEPES, 0.2 mM K-EGTA, 0.3 mM GTP, 2 mM Mg-ATP, 0.2 mM cAMP, 10 mM D-glucose, 0.06% rhodamine (and optional 0.15% biocytin)
• SMARTer Reaction Buffer: 1.1 μl of RNase Inhibitor (40 U/μl) mixed into 20.9 μl of Dilution Buffer (both supplied with the Clontech SMARTer – HV kit)
• Sample collection (lysis) buffer: 5.0 μl of SMARTer Reaction Buffer, 2.0 μl of nuclease-free water, and 1.0 μl of 1:250,000-diluted ERCC RNA-spike-ins mixed together
   ◦ Important: The specific dilution (amount) of ERCC spike-ins added should be carefully optimized for each experimental setup to ensure that the number of added spike-in molecules is in proportion to the number of cellular RNA molecules (i.e., the experimental sample is not overspiked).

## Stepwise Procedures

### Preparation of Poly-L-Ornithine/Laminin-Coated Glass Coverslips for Neuronal Culture

#### Etching Coverslips (Acid Wash)

Note: Glass coverslip manufacturers commonly apply a superficial layer of silicone coating to reduce sticking tendency. Because such coating affects proper adherence of the neurons to the coverslip, acid washing is performed to remove the silicone through etching of the glass surface, making the glass a better substrate for cellular attachment. Extreme caution should be exercised with the handling of the strong acids in the protocol (indicated below).

(1) In a fume hood, fill the sonicator bath with Milli-Q water to about a thumb below the max line.
(2) Place coverslips in a 1-l heavy duty borosilicate glass beaker. Note: If using 24-well tissue culture plates, use 12-mm diameter coverslips; if using 48-well plates, use 8-mm diameter coverslips.
(3) Fill glass beaker with Milli-Q water and swirl to rinse the coverslips. Then, pour off the water while being careful not to pour out the coverslips.
(4) Repeat the previous rinsing step three more times.
(5) Pour out as much of the remaining Milli-Q water as possible. Then, in the fume hood, fill the beaker with a sufficient volume (∼300 ml) of concentrated nitric acid to completely cover the coverslips and swirl. Caution: Nitric acid is extremely corrosive; handle with care and use appropriate personal protective equipment.
(6) Wrap the top of the beaker in Parafilm, place the beaker in the sonicator bath, and sonicate (in the fume hood) for 60 min. Note: The sonicator must remain in the fume hood because of the acid fumes.
(7) Dispose of the nitric acid in an appropriate waste collection bottle in the fume hood.
(8) Wash the coverslips three times with Milli-Q water; be sure to swirl during each wash.
(9) Pour off the water from the last wash, add a sufficient volume (∼300 ml) of concentrated hydrochloric acid to completely cover the coverslips, and swirl. Caution: Hydrochloric acid is extremely corrosive; handle with care and use appropriate personal protective equipment.
(10) Wrap the top of the beaker in Parafilm again, place the beaker in the sonicator bath, and sonicate (in the fume hood) for 60 min.
(11) Dispose of the hydrochloric acid in an appropriate waste collection bottle in the fume hood.
(12) Rinse the coverslips 20 (!) times or more until all the hydrochloric acid has been thoroughly removed.
(13) Pour off as much of the water as possible from the last rinse and transfer the coverslips from the beaker to a sterile (autoclaved) low-profile glass jar using a pair of forceps pre-cleaned with absolute [100% (v/v)] ethanol.
(14) Add absolute ethanol to the coverslips until sufficiently covered, and store the jar with the lid closed until ready to use the coverslips.

#### Transferring Coverslips to Sterile Tissue Cultureware

Important: Sterile technique must be used when working with etched coverslips. From this point on, all coverslip handling steps should be performed in a sterile tissue culture hood under aseptic conditions.

(15) In a biosafety hood, use a 25-ml serological pipette to fill up three sterile plastic reagent reservoirs with sterile (autoclaved) Milli-Q water.
(16) Use a pair of sterile forceps with delicate tips to remove coverslips from the glass jar with ethanol and transfer them to the first reagent reservoir with sterile Milli-Q for their first wash.
(17) Wash the coverslips another two times in sterile Milli-Q by transferring them from the first to the second, and then from the second to the third reservoir. Again, use the sterile forceps for this purpose.
(18) Use the sterile forceps to take out individual coverslips and transfer them to the wells of a tissue culture-treated cell culture plate. Important: Coverslips might sometimes stick together; ensure that only one coverslip is transferred and positioned flat on the bottom of each well.
(19) To each well containing a glass coverslip, add 500 (24-well plate) or 250 (48-well plate) μl of sterile Milli-Q water.
(20) With a plastic aspiration pipette connected to a vacuum aspirator, remove as much of the water from the coverslips as possible. Note: Using glass Pasteur pipettes for aspiration might scratch the coverslip surface. If plastic aspiration pipettes are unavailable, one can attach a sterile (autoclaved), non-filter pipette tip to a glass pipette to aspirate the water.
(21) Leave the plate (covered with lid) in the hood to dry for at least 2 h before coating.
(22) Once dried, if not used immediately, a plate with coverslips can be wrapped in plastic food wrap or Parafilm and stored outside the hood at room temperature until ready for coating.

#### Coating Coverslips With Poly-L-Ornithine and Laminin

(23) Dilute poly-L-ornithine (PORN) solution in sterile, tissue culture-grade water to a final concentration of 50 μg/ml.
(24) Add the diluted PORN solution to the etched coverslips in the multiple well culture plate. Recommended coating volumes are 300 and 200 μl per well for a 24- and 48-well plate, respectively. Gently swirl the plate to evenly spread the coating solution and ensure that the entire surface is covered.
(25) Incubate at room temperature for at least 2 h or seal the plate in plastic food wrap and incubate overnight in the refrigerator (2–8°C). Important: Ensure that the PORN solution does not evaporate.
(26) Dilute laminin in DMEM/F-12+GlutaMAX basal medium to a final concentration of 5 μg/ml. Important: Slowly thaw laminin solution on ice or overnight at 2–8°C, and do not vortex.
(27) Remove the PORN solution gently by aspiration. Important: Refrain from touching the coverslip as this might scratch the PORN coating.
(28) Perform a wash with PBS; i.e., (i) slowly add a small volume of PBS via the side of each well (while being careful not to disrupt the PORN coating on the coverslip), (ii) gently swirl to ensure the PBS covers the entire surface, and (iii) remove the PBS solution by aspiration.
(29) Repeat the PBS wash from above one more time.
(30) Perform one wash with DMEM/F-12+GlutaMAX basal medium.
(31) Add the diluted laminin solution (from Step 26) to the PORN-coated coverslips. Recommended coating volumes are the same as for PORN coating (see Step 24). Again, swirl to ensure that the coverslips are entirely covered by solution.
(32) Incubate at room temperature for at least 2 h or seal the plate in plastic food wrap and incubate overnight in the fridge (2–8°C). Important: Ensure that the laminin solution does not evaporate.
(33) If not used immediately for plating cells, the cultureware with PORN/laminin-coated coverslips should be sealed in food wrap or parafilm foil and stored at 2–8°C for up to 14 days. Cultureware stored in the cold must be equilibrated to room temperature for at least 30 min prior to seeding cells. When ready to plate cells, just aspirate the laminin solution; there is no need to wash the coverslips.

### Culturing Human Neuronal Cultures on Coverslips

The aim of this section is to explain how to generate human neuronal cultures on glass coverslips for Patch-seq experiments. We applied this protocol to human midbrain neuronal cultures derived from embryonic and induced pluripotent stem cells (ESCs/iPSCs) (Bardy et al., 2015, 2016), but the procedures described can be easily adapted to other types of neuronal tissue [e.g., forebrain/cortical neurons (Zeng et al., 2010; Shi et al., 2012)], or potentially even to non-neuronal tissues that are electrophysiologically interesting [e.g., cardiomyocytes (Ma et al., 2011; Davis et al., 2012)].

#### Plating Neural Progenitor Cells (NPCs) for Differentiation

Embryoid body formation (Kim et al., 2011; Boyer et al., 2012), monolayer culture (Chambers et al., 2009; Kim et al., 2011; Li et al., 2011), and stromal feeder co-culture (Kawasaki et al., 2000; Kim et al., 2006) protocols represent the three mainstream procedures for the neuronal induction of human pluripotent stem cells. Detailed protocols have been published for the production of a variety of brain region-specific cell types [e.g., dopaminergic (Lee et al., 2000; Boyer et al., 2012; Zhang et al., 2014), serotoninergic (Vadodaria et al., 2016; Xu et al., 2016), forebrain/cortical (Zeng et al., 2010; Shi et al., 2012; Muratore et al., 2014), and hippocampal dentate gyrus-like neurons (Mertens et al., 2015)] from stem/progenitor states as well as adult somatic tissue. The NPC induction protocol that is most relevant to the experimental model should be used. Here, we applied the protocol from Boyer et al. (2012) to derive NPCs for subsequent differentiation into midbrain-like neuronal cultures:

(34) Prepare six-well plates with hESC-qualified Matrigel coating according to manufacturer’s directions.
(35) Culture NPCs on the Matrigel-coated six-well plates at high densities (∼2–4 × 10^6^ cells per well). When passaging the cells, split at a ratio no higher than 1:3 about once every week.
   • Pitfall: If human NPC cultures do not grow or grow very slowly, the NPCs were split (subcultured) too sparsely. Do not split the NPCs at a ratio higher than 1:3 as passaging these cells at too low confluency initiates their spontaneous differentiation. Ensure all cell lines are regularly tested to be free of mycoplasma contamination, as its presence may also negatively affect cell growth and proliferation.
(36) Feed NPCs every other day with 2 ml/well culture volume of fresh, 37°C pre-warmed NPC medium.
(37) Validate the expression of NPC-specific marker proteins such as NESTIN, SOX2, and PAX6 in the NPCs.
(38) To plate NPCs onto PORN/laminin-coated coverslips for neuronal differentiation:
   (a) Aspirate medium and add 1 ml per well (six-well plate) of warm ACCUTASE. Incubate at 37°C for 5–10 min.
   (b) Gently finger-tap the bottom outside of the plate to ensure cell detachment.
   (c) Add 5 ml per well of DMEM/F12+GlutaMAX basal medium and gently transfer the dissociated cell suspension to a 15-ml conical tube. Important: Do not triturate the cell suspension, and try to minimize mechanical disturbances to the NPCs while aspirating and dispensing.
   (d) Centrifuge at 300 × *g* for 5 min to pellet the cells.
   (e) Remove the supernatant through aspiration while being careful not to disturb the NPC pellet.
   (f) Using a P1000 pipette, resuspend the cells in 1 ml of NPC medium per original well of NPCs. Important: Be very gentle when pipetting the cells as they are very sensitive to mechanical manipulation.
   (g) Count the number of viable NPCs in the Countess automated cell counter (preferable) or manual hemocytometer by trypan blue dye exclusion.
   (h) Remove laminin solution from the previously prepared plates containing PORN/laminin-coated coverslips.
   (i) Plate NPCs onto the PORN/laminin-coated coverslips at a density of approximately 75,000 (48-well plate) or 150,000 (24-well plate) cells per well in NPC medium.
   (j) Place the multiple well plate back in a humidified 37°C incubator with 5% CO_2_ for 24 h. To obtain a uniform adherence of NPCs across the coverslip surface, move the plate in a figure-eight motion a couple of times on the incubator shelf.

#### Differentiating and Maturing NPCs Into Functional Neurons

(39) After 24 h of recovery, gently replace half of the NPC medium with fresh, pre-warmed NDM to gradually initiate differentiation of the NPCs into midbrain-like neuronal subtypes.
(40) Continue to gently replace half of the medium in the wells with an equal volume of fresh, pre-warmed NDM three times a week, taking care to minimize fluid perturbations that may affect proper maturation of the neurons.
   • Pitfall: Occasionally neuronal cultures may detach from their coverslip after long period of time *in vitro*. To avoid this, when feeding the cultures, be sure to remove half of the old medium and add new medium very gently and slowly via the side of the well, being careful not to disturb the neuronal culture. Harsh feeding techniques may be causing too much medium turbulence on the cells. Also ensure that coverslips are etched and PORN/laminin-coated according to the protocol as sub-optimal coating can also cause detaching (Steps 1–33).
(41) If functional analyses (e.g., patch clamping and calcium imaging) are to be performed, ensure to allow sufficient time (∼2–3 weeks at a minimum) for the neurons to develop strong synaptic contacts. Proper neuron generation can be immunohistochemically validated by staining for neuron-specific class III β-tubulin (using TUJ1 antibody) and the mature neuronal marker MAP2AB.

### Decontamination of Patch-seq Equipment and Working Areas

Ribonucleases (RNases) are a type of enzyme that catalyzes the degradation of RNA. The minimal amount of RNA starting material in a single cell (∼10–30 pg) and the ubiquitous presence of several RNases require that an exceptional level of care must be taken to remove or inhibit any possible source of RNase contamination prior to and during lysis buffer preparation, single-neuron collection, and the pipetting of reverse-transcription mix reagents. In addition, due to the extreme sensitivity of the protocol, it is strongly recommended that the whole process up until the cDNA amplification step be carried out in a PCR-clean room (i.e., a room that never contains post-PCR amplification products) to avoid possible cross-contamination with cDNA from previous amplification reactions. The following steps describe how to clean all equipment and working areas in preparation for a Patch-seq experiment:

(42) With a spray bottle, apply RNase AWAY decontamination reagent to all equipment and working areas that will be touched with gloved hands during the experiment. Ensure the decontamination reagent contacts the entire surface by rubbing the wet area with a clean kimwipe, then use a fresh wipe to completely dry the surface. Note: Complete drying is important as residual RNase AWAY may degrade sample RNA.
   (a) For the sample collection area (electrophysiology rig), equipment to be cleaned includes stage platform, microelectrode holder, micromanipulator knobs, perfusion pump (outside surfaces), pipette puller (outside surfaces), and the desktop computer station (most importantly, the keyboard and mouse), plus any other areas frequently touched such as benches and (refrigerator and freezer) door handles.
   (b) For the sample processing area, equipment to be cleaned includes an RNase-free biosafety cabinet in which no DNA or PCR-amplified cDNA template has ever been introduced, a set of pipettes (P2, P10, P20, P100/P200, P1000) dedicated to RNA work, a 1.5-ml Eppendorf tube rack, two 0.2-ml PCR tube cooler racks, an ice bucket, and a laboratory marker pen. Note: The pipettes, tube racks, and lab marker are preferably left inside the clean room hood at all times and cleaned with RNase AWAY every day at the beginning of a new experiment. When cleaning the pipettes, ensure that no decontamination solution comes in contact with the gaskets or seals.

(43) UV sterilize all pipettes and tube racks inside the hood for 20–30 min prior to starting buffer preparation. Important: Do not UV sterilize enzymes or reagents.
(44) Once a week, decontaminate frequently touched surfaces such as benchtops, the inside surfaces of the PCR-clean hood, and door handles with a 10% (v/v) solution of bleach (sodium hypochlorite). This step eliminates contaminating RNA and DNA templates. Note: To prevent corrosion, metal surfaces should always be rinsed with dH_2_O following sodium hypochlorite decontamination.

### Preparation of Lysis Buffer for Single-Neuron Collection

Important: This section details how to prepare lysis buffer for sample collection using reagents from the SMARTer Ultra Low Input RNA for Illumina Sequencing – High Volume kit (Clontech, #634828). The Smart-seq method on which this kit is based has recently made several improvements that have resulted in optimized reagents and protocols for later version SMARTer kits (i.e., SMARTer v3 and SMART-Seq v4). Always follow the specific manufacturer’s instructions that come with the kit that is used.

(45) In the decontaminated PCR clean hood, prepare a stock solution of SMARTer Reaction Buffer by mixing 20.9 μl of Dilution Buffer with 1.1 μl of RNase Inhibitor (40 U/μl). Important: Do not vortex the RNase Inhibitor enzyme; just finger-flick the tube to mix the solution and spin down briefly in a microcentrifuge. Keep RNase Inhibitor and prepared SMARTer Reaction Buffer on ice at all times.
(46) Prepare sample collection (lysis) buffer for all cell samples and any negative or positive controls by combining the reagents in the table below in RNase-free 0.2-ml PCR tubes (one tube for each sample). Before dispensing reagents, ensure that the tubes are pre-chilled in a clean PCR cooler rack. Important: Thaw ERCC spike-in dilution on ice, and mix all components gently yet thoroughly before and after combining by finger-flicking and spinning down in a microcentrifuge. Important: The specific dilution (amount) of ERCC spike-ins added should be carefully optimized for the specific experimental setup to avoid overspiking the experimental sample.

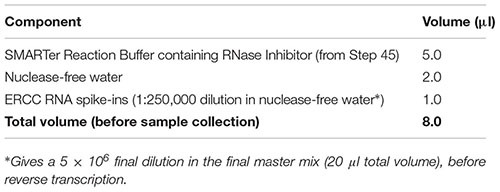

(47) Keep the PCR tubes with lysis buffer in the PCR cooler rack on ice (in a decontaminated ice bucket) until ready for single-neuron collection.

### Transferring Cells to Patch Clamping Setup

(48) In the tissue culture hood, transfer an individual coverslip to which neurons are attached to a well of a new 24-/48-well culture plate:
   (a) Gently aspirate half of the NDM covering the neurons and dispense into a well of a new 24-/48-well plate.
   (b) Using a pair of sterile forceps with ultrafine tips, carefully transfer the coverslip with neurons attached into this medium on the new plate. Note: To avoid damaging the neuronal culture on the coverslip, grasp the coverglass only along the very outer edge.
   (c) Cautiously transport the plate containing the single coverslip with neurons to the microscope.
   (d) At the microscope, using a clean pair of forceps and applying the same technique as described above, gently transfer the coverslip into the bath imaging chamber filled with freshly supplied medium. Lightly press down on the edge of the coverglass with the forceps to fully submerge the coverslip in the pre-warmed perfusate medium.

### Electrophysiological and Morphological Neuronal Characterization

**FIGURE 1:**
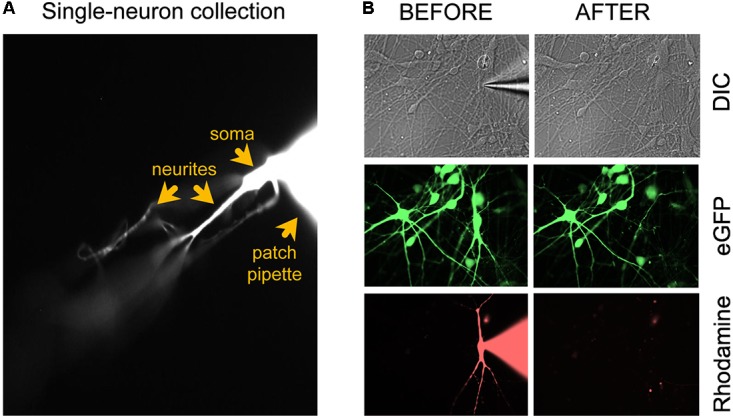
Microscopic evaluation of the neuron isolation procedure. **(A)** After applying additional negative pressure to establish a stronger seal between neuron and patch electrode, the neuron should remain attached to the patch electrode tip when the pipette is retracted for cell collection. **(B)** Confirmation of successful removal of the entire neuron from the coverslip after the collection procedure. Figure adapted from Figure 4a in Bardy et al. (2016) (^®^2016, Springer Nature).

(49) Fill patch-clamping pipettes with intracellular recording solution containing a rhodamine dye (see the “Reagent Setup” section). The electrophysiological recordings should last ∼15–30 min to allow enough time for the rhodamine (or alternative dye) to diffuse throughout the distal parts of the dendrites and axon (**Figure 1**). Perform whole-cell patch-clamp electrophysiology according to lab-preferred protocol. The specific protocols that we used to patch clamp *in vitro* human neurons derived from pluripotent stem cells are published in detail elsewhere (Bardy et al., 2015, 2016).

(50) At the end of the electrophysiological recording, maintain the cell in voltage clamp while taking fluorescent photos (40×) of the live neuron. Take images covering the largest field of view possible (>150 μm radius). Move the field of view gently without losing the patch. Use extra care when imaging above the patch pipette. Stitch the images and reconstruct/analyze the neuronal morphology offline with the appropriate software (e.g., Neurolucida). Including biocytin in the internal solution will allow you to fix and co-stain the patched neurons with immunocytochemistry markers, as an alternative to RNA-seq.

### Micropipette Isolation of a Single Neuron After Patch Clamping

Once the patch clamping and imaging are completed (<30 min), apply negative pressure to the patch pipette and slowly withdraw the neuron from the rest of the culture. The entire neuron, including the soma, dendrites, and axon (**Figure 1**), must be immediately transferred into the sample lysis buffer in an RNase-free collection tube:

(51) Apply negative pressure to the patch pipette to establish a strong seal with the neuron (-0.15 PSI).
(52) Carefully retract the glass micropipette from the chamber bath while visualizing the rhodamine-tagged cell and pipette under the binocular of the microscope (40×). Adjust the focus up and down to make sure that long dendrites are coming along with the soma. The neuron should remain attached to the tip of the electrode (**Figure 1A**). Important: confirm successful removal of the entire neuron from the coverslip by taking 40× images before and after removal (**Figure 1B**). The neuronal culture may be tagged with a GFP reporter (e.g., lentiviral vector) and the single patched neuron with rhodamine. Before patch clamping, collect GFP and DIC images. During patching, collect DIC and rhodamine images. After single-neuron collection, collect DIC, GFP, and rhodamine images. In the event that collection of the entire neuron is ambiguous, e.g., due to rupture of the cell’s elaborate neurites, the sample should be discarded from further processing.
   • Pitfall: If the neuron does not want to lift off from the coverslip when retracting the patch pipette, despite applying negative pressure, the neuronal culture might be too dense and/or the cell to be collected is too big (relative to the patch electrode tip) or has very long and complex dendritic arborization. Pull a new patch pipette with a slightly larger electrode tip to collect the larger neuron. If neuron isolation remains difficult after multiple collection attempts, consider plating a lower number of NPCs onto the PORN/laminin-coated coverslips for neuronal differentiation to obtain cultures that are less dense.
(53) Transfer the entire neuron, including its processes, into the sample collection buffer of the PCR collection tube:
   (a) Carefully remove the micropipette, with the neuron attached to its tip, from the pipette holder.
   (b) Transfer the neuron into the 8.0 μl of lysis buffer by submerging the patch electrode in the buffer and gently breaking its glass tip along the inside wall of the PCR collection tube. Ideally, the sharp point of the micropipette should be completely released into the buffer.
   (c) Insert a syringe into the top of the micropipette. Forcefully push down the plunger to effectively expel any remaining liquid contents out of the pipette. Gently remove the plunger and repeat fluid ejection one more time.
(54) Close the collection tube and spin in a microcentrifuge for 1 min to bring the contents to the bottom of the tube. Immediately place the tube back in the PCR cooler rack and proceed to cDNA synthesis of polyadenylated transcripts using Smart-seq-based chemistry. Because RNA is less stable than DNA, it is best to reverse transcribe each sample as soon as possible after collection. To accomplish this task, it is strongly recommended to have an experienced electrophysiologist work closely together with a molecular biologist, where the electrophysiologist is responsible for the patch clamping and sample collection, and the molecular scientist for performing immediate cDNA synthesis *post* sample collection. We do not recommend keeping collected samples on ice for extended periods of time (i.e., more than 1–2 h). Likewise, it is not recommended to freeze collected single cell samples prior to mRNA reverse transcription, as any freeze–thaw cycle will greatly accelerate degradation of the single-cell RNA. After cDNA synthesis, amplification and purification have been performed, the cDNA is stable and can be stored at ≤-20°C for up to 6 months before analysis or the preparation of libraries for RNA sequencing.

### Reverse Transcription of the Single-Cell RNA

Note: The Smart-seq method on which this kit is based has recently made several improvements that have resulted in optimized reagents and protocols for reverse transcription in later version SMARTer kits. Always follow the specific manufacturer’s instructions that come with the kit that is used.

(55) Check the sample for the presence of air bubbles. Remove any bubbles by finger-flicking the side of the PCR tube and spin down briefly (∼5 s) in a microcentrifuge to collect all contents at the bottom.
(56) Place the sample on a clean PCR cooler rack in the PCR clean hood.
(57) Add 1 μl of 3′-SMART CDS Primer II A (24 μM), mix the contents by gently vortexing at low speed, and spin the tube briefly in a microcentrifuge. Important: If necessary, remove any bubbles before spinning down by tapping the side wall of the tube.
(58) Denature RNA secondary structure by incubating the sample in a hot-lid thermal cycler using the following program:

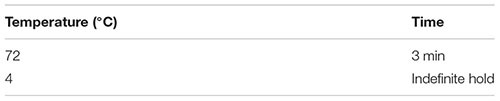


Important: The next steps are critical for cDNA synthesis and should not be delayed once this 3-min incubation step is complete. To be able to quickly start the reverse transcription, prepare reverse transcription master mix (Step 59) while the sample is incubating.

(59) While the sample is incubating in the thermal cycler, prepare reverse transcription master mix by combining the reagents from the table below in the order in which they are listed, at room temperature. Note: Add a 10% extra to the volumes listed to account for pipetting errors. Important: Ensure that all reagents are thawed completely and mixed well before addition to the mix. Do not vortex the reverse transcriptase enzyme; just finger-flick the tube to mix the solution and spin down briefly in a microcentrifuge. Add the reverse transcriptase enzyme just prior to use, mix gently yet thoroughly on the vortex, and briefly spin down the tube.

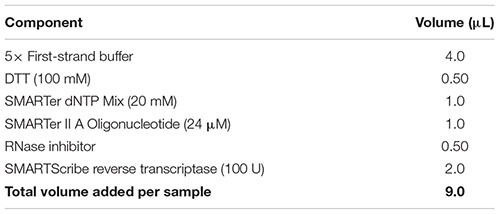

(60) Add 9 μl of the reverse transcriptase master mix to the denatured sample as per the following table. Gently pipette up and down five times to mix all components with the sample. Remove any bubbles by tapping the tube, and spin down for 5 s in a microcentrifuge to collect all contents at the bottom.

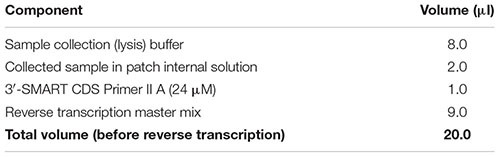

(61) Incubate the sample in a hot-lid thermal cycler using the following program:

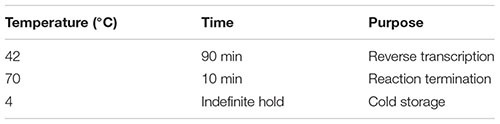

(62) Proceed immediately to amplification of the full-length cDNA.

### Purification and PCR Amplification of Full-Length cDNA

Note: The Smart-seq method on which this kit is based has recently made several improvements that have resulted in optimized reagents and protocols for cDNA amplification in later version SMARTer kits. Always follow the specific manufacturer’s instructions that come with the kit that is used.

(63) Bring AMPure XP magnetic beads to room temperature and vortex well to completely resuspend the beads in the bead/buffer solution.
(64) Add 36 μl of resuspended bead solution to the sample (1.8:1 vol:vol ratio of beads:sample). Adjust the pipettor to 56 μl and mix well by pipetting up and down the entire volume at least 10 times.
(65) Incubate at room temperature for 8 min to let the cDNA bind to the beads.
(66) Spin down briefly to collect liquid from the sides of the tube.
(67) Place the sample on the DynaMag-PCR magnetic separation device for 5 min until the solution is clear and the beads are collected at the side of the PCR tube.
(68) While the sample is sitting on the magnetic separation device, prepare amplification master mix by combining the reagents in the following table in the order in which they are listed, and store on ice.
   Note: Add a 10% extra to the volumes listed to account for pipetting errors. Important: Ensure that all reagents are thawed completely and mixed well before addition to the mix. Do not vortex the polymerase mix; just finger-flick the tube to mix the solution and spin down briefly in a microcentrifuge. Mix gently yet thoroughly on the vortex after combining all components, and briefly spin down the tube to collect contents at the bottom.

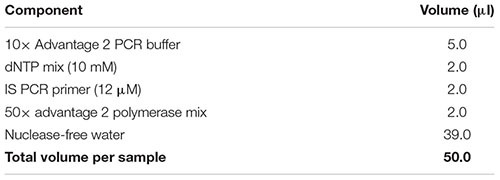

(69) While the sample is sitting on the magnetic separation device, carefully pipette out and discard the clear solution without disturbing the magnetic beads. This removes unincorporated nucleotides, primers, and small [<100 base pairs (bp)] cDNA fragments.
(70) Spin down briefly to collect liquid from the sides of the tube.
(71) Place the sample back on the magnetic separation device for an additional 2 min or longer until the beads are completely separated from the liquid.
(72) Using a P10 pipettor, carefully remove as much residual liquid as possible while taking care not to touch, disturb, or aspirate the beads.
(73) Add 50 μl of amplification master mix (from Step 68) to the DNA bound to the beads. Mix well and briefly spin down.
(74) Incubate the sample in a hot-lid thermal cycler using the following program. Important: It is strongly recommended to use a thermal cycler different from the one used for RNA denaturation and reverse transcription to prevent cross-contamination of new samples with aerosolized cDNA from previous amplification reactions. If it is not possible to use a different thermal cycler for the cDNA amplification, ensure that you thoroughly clean the entire PCR machine after each amplification run with RNase AWAY decontamination solution, which also eliminates DNA.

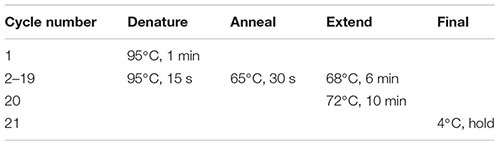

   Note: The amplified, unpurified cDNA can be stored at ≤-20°C for up to 3 months before continuing with PCR purification.

### Purification of PCR-Amplified cDNA

Important: Transfer the amplified cDNA sample from the pre-PCR area to a post-PCR laboratory (e.g., the general lab). All downstream steps should be performed in the post-PCR location with a set of dedicated pipettors to avoid cross-contaminating newly collected samples with PCR-amplified cDNA.

(75) Bring AMPure XP magnetic beads to room temperature and vortex well to completely resuspend the beads in the bead/buffer solution.
(76) Add 90 μl of resuspended beads to a new RNase/DNase-free microcentrifuge tube. Transfer the PCR-amplified cDNA sample (including the magnetic beads from the previous purification step) to this 90 μl of bead solution and mix well by pipetting the entire volume up and down 10 times.
(77) Incubate at room temperature for 8 min to let the cDNA bind to the beads.
(78) Place the sample on the DynaMag-2 magnetic separation device for 5 min until the solution is clear and the beads are collected at the side of the tube.
(79) While the sample is sitting on the magnetic separation device, carefully pipette out and discard the supernatant.
(80) With the sample still sitting on the magnetic separation device, add 200 μl of freshly made 80% (v/v) ethanol (molecular biology grade ethyl alcohol diluted with nuclease-free water) to the beads. Wait for 30 s and then carefully pipette out and discard the ethanol solution without touching or disturbing the beads.
(81) Repeat the ethanol wash from Step 80 one more time.
(82) Spin the sample for 10 s in a microcentrifuge to collect residual ethanol from the sides at the bottom of the tube.
(83) Place the sample back on the magnetic stand for 30 s and pipette out all the residual ethanol.
(84) Leave the sample, with the lid closed, at room temperature for 3–5 min until the bead pellet appears dry (i.e., tiny cracks have become visible in the pellet). Be sure to dry the pellet enough, as any residual ethanol from under-dried sample will reduce the final cDNA yield. However, do not overdry the pellet as this will make it difficult to rehydrate and resuspend the beads in SMARTer purification buffer.
(85) Remove the sample from the magnetic stand, add 12 μl of SMARTer purification buffer to cover the beads, and incubate for 2 min to rehydrate.
   • Pitfall: If the magnetic beads are difficult to resuspend, the beads are over-dried. Do not wait for all sample pellets to appear completely dry before adding SMARTer purification buffer; over-drying of the beads can be avoided by purifying samples in smaller batches. cDNA from over-dried beads may still be recovered by covering the beads in purification buffer for an extended amount of time (>2 min).
(86) Pipette up and down 10 times to resuspend the beads and elute the cDNA.
(87) Place the sample back on the magnetic separation device and incubate for 2 min until the beads are collected to the side of the tube and the solution is clear.
(88) With the sample sitting on the magnetic stand, transfer the clear supernatant (∼12 μl) to a non-stick, RNase-free microfuge tube.

Note: The purified amplified cDNA can be stored at ≤-20°C for up to 6 months before continuing with QC analysis and library preparation.

### QC of Single-Neuron cDNA

**FIGURE 2:**
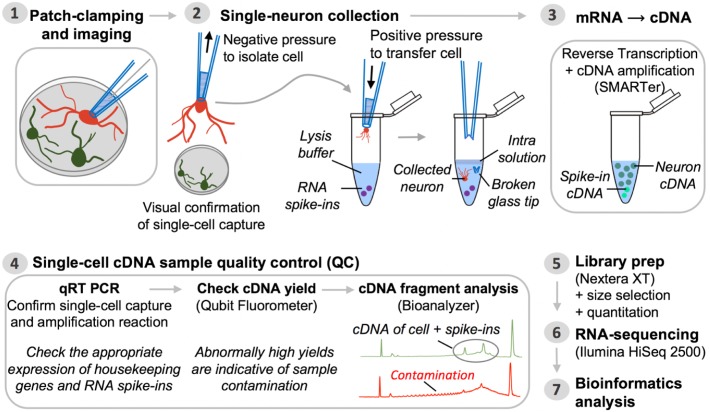
Pipeline for single-neuron Patch-seq experiments. Step 1: A single neuron is characterized at the functional and morphological level by whole-cell patch-clamp electrophysiology and imaging. Step 2: Negative pressure is applied to the patch pipette used for electrophysiological recording, after which it is retracted from the chamber batch with the neuron still attached. The entire neuron, including its processes, is immediately transferred into lysis buffer by breaking the glass patch pipette tip along the inside wall of an RNase-free collection tube. Successful cell capture is always confirmed by microscopic evaluation (comparison of before and after pictures). Step 3: Single-neuron mRNA, along with external reference transcripts (ERCC RNA spike-ins) present in the lysis buffer, is reverse transcribed and the cDNA is PCR-amplified using SMARTer chemistry. Step 4: The synthesized cDNA is subjected to a series of QC steps based on three assays: (i) expression profiling of common housekeeping genes and RNA spike-ins by quantitative real-time PCR; (ii) fluorometric quantitation of cDNA yield (Qubit); and (iii) qualitative analysis of cDNA fragment profiles and contamination check (Bioanalyzer). Inability to detect expression of housekeeping genes while detecting expression of ERCCs is a sign of failed neuron capture, whereas inability to detect ERCCs signifies failed SMARTer cDNA synthesis. Contaminated samples are characterized by abnormally high cDNA yields that appear on the fragment analyzer as a broader peak with jaggedness or multiple peaks in the electropherogram (see also **Figure 3**). Steps 5 and 6: Libraries for RNA-seq are prepared from samples passing all QC analyses and are sequenced on an Illumina HiSeq 2500 platform. Step 7: Bioinformatics processing and analysis of the resulting RNA-seq data enable identification of unique genetic signatures associated with specific neuronal (sub)types.

Thorough sample QC ensures that potentially spurious captures and/or contaminated samples can be readily identified and filtered out prior to proceeding with expensive transcriptome profiling. This section details a general workflow for the QC analysis of cDNA samples generated from captured single neurons using Smart-seq chemistry (**Figure 2**). Note: The specific results obtained may vary slightly with the use of reagent kits that employ newer Smart-seq chemistry; the steps below demonstrate a general pipeline of QC analysis to be performed on single-cell cDNA. Always consult the user manual of your kit for specific recommendations.

**FIGURE 3:**
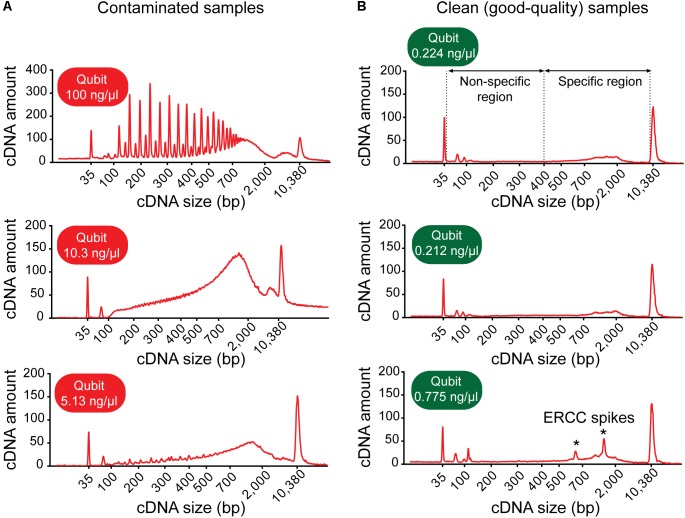
Example electropherograms of contaminated versus clean (sequencing-quality) cDNA samples generated from single neurons. **(A)** Samples with high cDNA yields are typically characterized by distinctive features of the cDNA fragment profile indicative of contamination: multiple sharp peaks (*top* figure) or an early rising peak with increased cDNA amount at lower base-pair sizes and a “jagged” pattern (*middle* and *bottom*). **(B)** Samples generating good-quality sequencing data have a shallow peak with a maximum at ∼2,000 bp (range ∼400–9,000 bp, *specific region*), no jaggedness, and no cDNA product at lower base pair size (*non-specific region*). Addition of ERCC RNA spike-in controls to the sample collection buffer may result in the appearance of additional peaks (indicated by asterisks).

(89) Profile the expression levels of at least two widely expressed housekeeping genes (e.g., *GAPDH* and *ACTB*) with standard quantitative real-time PCR (qRT-PCR):
   (a) Order pre-formulated TaqMan Gene Expression Assays (primers) for your housekeeping genes to be assayed. We selected human beta actin (ACTB; Applied Biosystems, 4333762T) and GAPDH (Applied Biosystems, 4352934E) as endogenous controls.
   (b) In a non-stick RNase-free tube, make a 1:5 dilution of single-neuron cDNA sample by pipetting 2.0 μl of sample into 8.0 μl of nuclease-free water. Mix well by brief vortexing and spin down the sample in a microcentrifuge.
   (c) Prepare a Master Mix on ice for each housekeeping gene to be assayed. Per 10-μl reaction in one well of a 384-well plate, use 5.0 μl of 2× TaqMan Gene Expression Master Mix, 0.50 μl of 20× TaqMan Gene Expression Assay/Primer, and 4.0 μl of nuclease-free water. Mix solutions by vortexing and centrifuge briefly to spin down contents and eliminate air bubbles. Note: Make 10% more than the overall number of reactions required to account for pipetting errors.
   (d) Per reaction, combine 9.50 μl of the appropriate Master Mix and 0.50 μl of 1:5-diluted cDNA in a well of a 384-well plate (10 μl total volume). Setup at least two technical replicates per sample to ensure experimental reproducibility.
   (e) Briefly centrifuge the reaction plate to spin down contents and eliminate air bubbles.
   (f) Run the plate on a qRT-PCR instrument.
      • Pitfall: If qRT-PCR analysis of *ACTB* and *GAPDH* housekeeping genes reveals no or very low (Ct > 30) expression, it is likely that the cell was lost from the pipette tip during retraction of the patch electrode from the chamber bath, or during its transfer into the PCR tube. Alternatively, RNA from a successfully captured cell degraded due to RNase contamination of the sample or improper sample handling. Keep single-cell samples on ice at all times (unless indicated otherwise) and proceed with reverse transcription immediately after cell collection. Do not freeze samples before reverse transcription and cDNA amplification have been completed. Clean all lab benches, pipettors and equipment thoroughly with RNase AWAY decontamination solution every day, before starting experiments.
(90) Profile the expression levels of a high, medium, and low abundant ERCC spike-in RNA standard (included in the sample collection buffer as “ERCC RNA Spike-In Mix”) with standard qRT-PCR using the same protocol as described above. Pre-designed TaqMan Gene Expression Assays for these external controls are available from Thermo Fisher Scientific.
(91) Quantitate the yield of each cDNA sample using the Qubit Fluorometer with the dsDNA HS Assay Kit according to the manufacturer’s instructions. Use 1.0 μl of sample for measurement. Note: Contaminated samples are commonly characterized by an abnormally high cDNA concentration (typically >3 ng/μl with 18-cycle PCR amplification). Whether or not a sample with high cDNA yield is truly contaminated can be confirmed by analysis of its cDNA fragment profile/size distribution (detailed below).
(92) Assess the quality of each sample by subjective inspection of the cDNA fragment profile:
   (a) Run 1 μl of single-cell cDNA sample on a HS DNA Chip with the Agilent 2100 Bioanalyzer according to the manufacturer’s instructions. Note: Prior to sample analysis, make sure that the HS DNA Ladder displays a flat baseline and clearly resolved peaks (*n* = 15), and it correctly identifies the Upper and Lower Marker.
      • Pitfall: If the baseline of the Bioanalyzer trace does not appear flat/straight, the magnetic beads have carried over into the cDNA sample: Any DNA bound to them will bind dye and affect the baseline fluorescence. Place the cDNA sample onto the magnetic stand for 5 min to attract any beads onto the tube wall. Using a small pipette tip, slowly transfer bead-free solution to a new microfuge tube while taking care not to disturb any beads into solution.
      • Pitfall: If the cDNA fragment profile shows a large peak following the Bioanalyzer Lower Marker (<100 bp), carryover of primers or primer dimers has occurred. During cDNA purification, ensure that ethanol wash solutions are always prepared fresh and completely removed and that the magnetic beads are sufficiently dried to enable complete evaporation of residual ethanol before resuspension.
   (b) Inspect the electropherogram for signs of contamination. Contaminated samples can be identified by distinctive electropherogram features: (i) an abnormally high yield, (ii) a broader than normal peak with increased cDNA amounts at lower bp sizes, and/or (iii) a “jagged” pattern or multiple peaks (**Figure 3A**). Samples with features indicative of contamination should be excluded from downstream library preparation and RNA-sequencing analysis.

   (c) Check for the presence of specific cDNA product in the range of 400–9,000 bp. Note: A sample generating good quality data is typically characterized by a shallow peak with a maximum at ∼2,000 bp and has no cDNA product at lower bp sizes (**Figure 3B**).

### Library Preparation

#### Tagmentation

(93) Dilute the cDNA sample to a concentration of 0.20 ng/μl in nuclease-free water.
(94) Perform tagmentation with the Nextera XT DNA library preparation kit according to manufacturer’s instructions with the following reagent quantities:

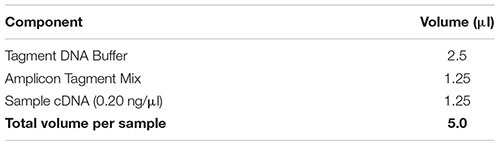

(95) Place the sample in a thermal cycler and incubate as follows with a heated lid:

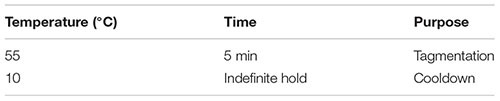

(96) As soon as the sample reaches 10°C, immediately add 1.25 μl of Neutralize Tagment Buffer to the tagmented sample to stop the tagmentation reaction. Pipette to mix, centrifuge for 1 min, and incubate at room temperature for 5 min.

#### Library Indexing and Amplification

(97) Add 1.25 μl each of Index Primer 1 (N7XX) and Index Primer 2 (S5XX) to the tagmented cDNA. Important: Ensure that each sample is given a unique combination of Index 1 and Index 2 primers.
(98) Add 3.75 μl of Nextera PCR Master Mix (NPM). Pipette to mix, centrifuge for 1 min, and perform PCR amplification using the following program with heated lid:

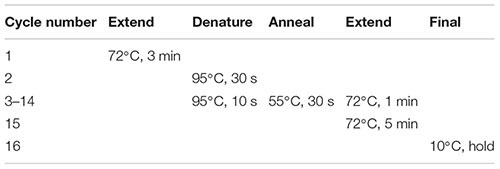


Note: PCR-amplified cDNA libraries can be stored at ≤-20°C for up to 6 months before size selection, pooling, and sequencing.

#### Size Selection and Library Pooling

(99) Run PCR-amplified cDNA libraries on a 1.5% agarose gel in Tris-borate/EDTA (TBE) buffer, stain with SYBR Gold nucleic acid stain (1:10,000 in TBE), and gel-excise the ∼300–400 bp size region of each library.
(100) Purify gel-excised library fragments with the Wizard SV Gel and PCR Clean-Up System according to manufacturer’s instructions, eluting the size-selected cDNA in 40 μl of nuclease-free water.
(101) Concentrate the size-selected library samples: Decrease the sample volume to 13–14 μl by speedvacuum centrifugation.
(102) Quantify the mean concentration and determine the mean size of each size-selected library using, respectively, the Qubit Fluorometer and Bioanalyzer according to manufacturer’s instructions.
(103) Pool equimolar amounts of uniquely barcoded libraries together to create a pooled library sample. Note: The pooled cDNA library can be stored at ≤-20 for up to 6 months prior to sequencing.
(104) Quantify the concentration of the pooled library by qPCR (KAPA Library Quantification Kit for Illumina Platforms according to manufacturer’s instructions).

### Sequencing

(105) Sequence the pooled library sample on a HiSeq 2500 sequencer according to manufacturer’s directions.

## Anticipated Results

Using the patch-pipette micromanipulation technique detailed here, the likelihood of successful isolation of a single neuron from the culture substratum (as confirmed by microscopic evaluation; **Figure 2**, Step 2) is approximately 50%. It should be noted, however, that the actual success rate of removing a patch-clamped neuron from a coverslip may vary somewhat from laboratory to laboratory and even from culture to culture depending on the type of neurons grown, their density on and adherence to the coverslip, their thickness and size, and dendritic complexity as well as the experimenter’s experience or proficiency with the isolation technique. Of all the samples captured (visually confirmed) and reverse transcribed, about 50% pass all QC at the cDNA level (**Figure 2**, Step 4), thus bringing the total success rate of obtaining sequencing-ready libraries from electrophysiologically characterized neurons to ∼25%. Following the protocol detailed here, 56 single neurons derived from healthy pluripotent stem cells (ESCs/iPSCs) were isolated and subjected to whole-transcriptome mRNA-seq profiling after passing all sample QC (see Bardy et al., 2016).

### Housekeeping Gene Expression

**FIGURE 4:**
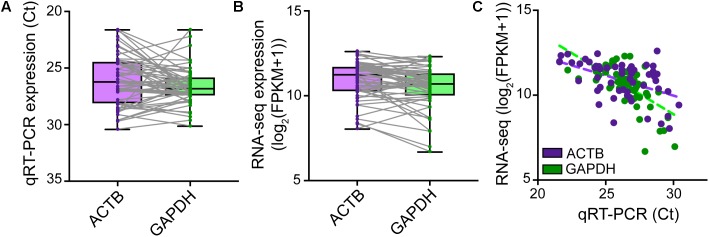
Natural variation in expression of housekeeping genes in single neurons. TaqMan quantitative real-time PCR was performed on SMARTer cDNA generated from *n* = 56 single human neurons successfully isolated for Patch-seq to profile the expression levels of *ACTB* and *GAPDH* housekeeping genes. *ACTB* and *GAPDH* were readily expressed though at variable levels from cell to cell as revealed by both qRT-PCR **(A)** and RNA-sequencing **(B)**. **(C)** Single-neuron expression of *ACTB* and *GAPDH* housekeeping genes is highly correlated between qRT-PCR and RNA-sequencing measurements.

Although microscopic evaluation is used to confirm successful removal of the neuron from the coverslip, a cell can be lost from the pipette tip upon its retraction from the chamber bath due to the surface tension present at the perfusate–air interface. A quantitative real-time PCR assay profiling the expression of a few housekeeping genes should provide comprehensive information as to whether a cell was successfully collected into the PCR tube. qRT-PCR analysis of cDNA reverse transcribed and amplified from effectively captured neurons reveals good expression of the housekeeping genes *ACTB* and *GAPDH* (Ct values ≤ 30), though at variable levels from cell to cell (**Figure 4A**). Samples with no or very low (Ct > 30) expression of housekeeping genes can be considered spurious. Such samples very likely represent failed captures, and therefore we recommend excluding them from downstream sequencing analysis. We readily detected expression of housekeeping genes with RNA-seq in cells that passed all QC checks in our pipeline (**Figure 4B**), and RNA-sequencing expression correlated highly with the qRT-PCR measurements (**Figure 4C**).

### Expression of RNA Spike-in Standards

**FIGURE 5:**
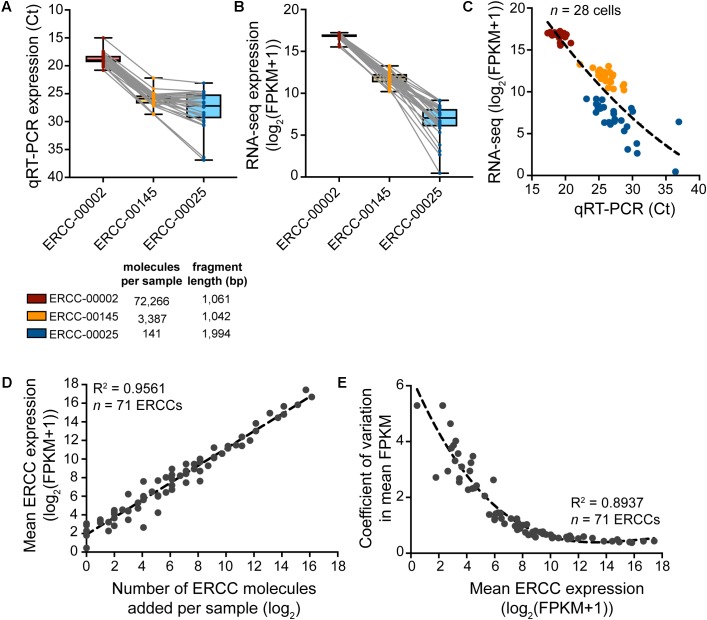
Expression analysis of ERCC RNA spike-in controls to assess technical performance of SMARTer cDNA preparation. **(A–C)** Expression of a high-, medium-, and low-copy ERCC standard in *n* = 28 spiked cells measured by qRT-PCR and RNA-seq. Variability in expression increases with a decrease in absolute ERCC copy number. **(D)** Measured expression of ERCC RNA spike-ins as a function of the number of actual molecules added to each sample. The very small amount (∼10 pg) of RNA in a single cell requires a significant dilution of the ERCC stock to avoid overspiking of the experimental sample with exogenous transcripts. With a 1:5,000,000 dilution in the final master mix before reverse transcription, 73 ERCCs were present in at least one copy number, 71 of which were detected by RNA-seq across samples (*n* = 28). **(E)** The variation in measured ERCC expression levels is highest for low expressed (i.e., low abundant) ERCCs.

To assess the sample-specific technical performance of the mRNA reverse transcription and cDNA amplification reactions, synthetic reference transcripts (ERCC RNA spike-ins) should be mixed into the sample before cell collection and their expression analyzed after cDNA preparation (**Figure 5**). Expression profiling of three different ERCC transcript species with qRT-PCR and sequencing reveals that sample-to-sample variation in expression is smallest for the highest copy number ERCC transcript but is increased for transcript species present at lower abundances (**Figures 5A–C**). Confirming the efficacy and non-specificity of the cDNA synthesis and amplification reactions, the absolute transcript abundances of the ERCC standards correlate very strongly with their normalized expression reads as measured by RNA-sequencing (**Figure 5D**). The dispersion in the variation in mean expression levels of ERCCs decreases with increased expression (and hence, abundance) of ERCC transcripts (**Figure 5E**), in line with the qRT-PCR data. This is to be expected because there is less technical variability in the wet-lab addition as well as the detection (RNA-sequencing) of ERCC transcripts of higher abundance. All in all, qRT-PCR analysis of the ERCC spike-ins represents a useful tool to quickly assess the quality of the single-cell transcriptome preparation prior to sequencing.

As a word of caution, the concentration of spike-in controls such as ERCCs added to the sample collection buffer should be carefully adjusted to ensure that the majority of sequencing reads originate from the experimental sample and not from the added spike-ins. The ERCC spike-in stock should be diluted in such a way that the number of spike-in molecules added to each sample is in proportion to the number of cellular RNA molecules. Our 1:250,000 dilution of ERCC stock gives a final concentration of 1:5,000,000 in the final master mix before reverse transcription and results in the detection of 71 out of 92 ERCCs. One has to optimize the final concentration of ERCCs for the specific experiment, depending on factors such as the size of the cells and the number of transcripts typically expressed in the cell type to be collected.

### Quantity and Quality Analysis of Single-Cell cDNA

**FIGURE 6:**
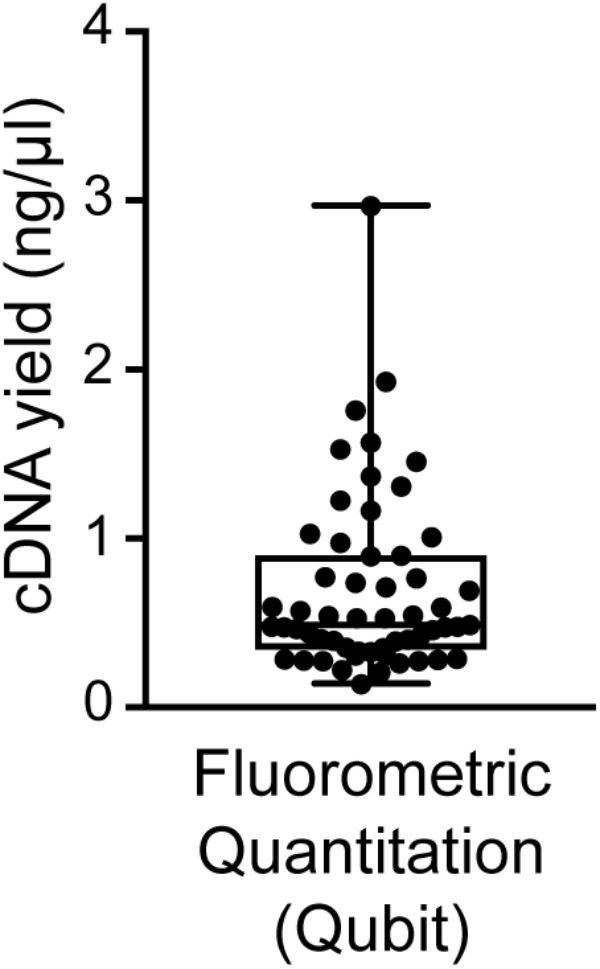
Distribution of cDNA yields of sequencing-quality single neuron samples. Reverse transcription and 18-cycle PCR amplification of cDNA from single neurons using SMARTer resulted in yields of cDNA in the range of 0.14–2.97 (mean 0.70 ± 0.53) ng/μl. We found samples with substantially higher yields to be frequently contaminated (see also **Figure 3**).

We found good-quality samples suitable for sequencing to have a cDNA concentration in the range of 0.14–2.97 (mean 0.70 ± 0.53) ng/μl (**Figure 6**). This represents the typical concentration range of cDNA that is obtained using 18-cycle PCR amplification with Smart-seq. Beware that newer kits employing improved Smart-seq chemistry likely produce higher cDNA yields. Additional inspection of the fragment profiles (Agilent 2100 Bioanalyzer) of various amplified cDNA samples shows that many samples with abnormally high cDNA yields (>3 ng/μl) are characterized by signs of contamination: a broader than normal peak with increased cDNA amounts at lower bp sizes, and a pattern of multiple peaks or “jaggedness” in the electropherogram (**Figure 3A**). In contrast, good-quality (clean) samples show the presence of specific cDNA product in the 400–9,000 bp range (with a maximum at ∼2,000 bp), no jaggedness, and no low bp-sized cDNA product (**Figure 3B**). Thus, both a quantitative and qualitative assessment of Smart-seq-synthesized cDNA prior to library preparation and sequencing can be used to identify, and filter out, cell samples with a high likelihood of generating low-quality data.

### Timing of the Protocol

**FIGURE 7:**
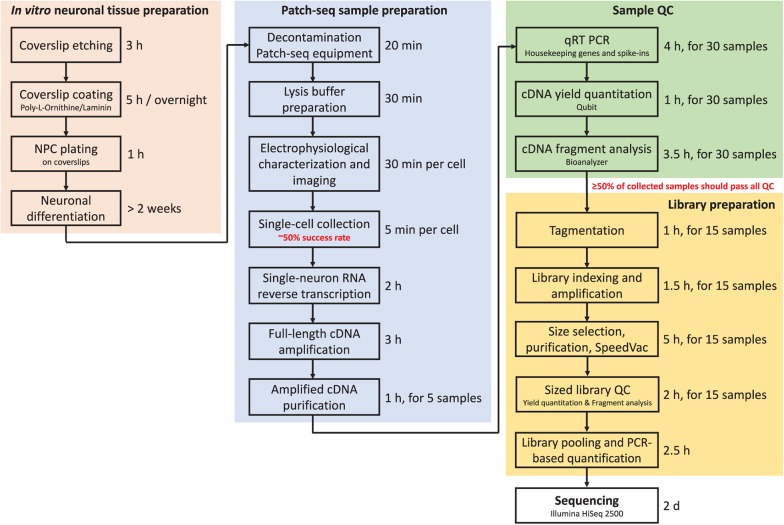
Flow chart of the Patch-seq procedure indicating timing for each step.

Throughput is the major limitation of Patch-seq as it takes time to patch, image, and collect a single cell. In our experience, an experienced electrophysiologist and molecular biologist working closely together can successfully record, collect, and process (reverse-transcribe and amplify) 3–4 cells per day (out of six to eight total cells patched). This amounts to 15–20 cells per week that can be collected and subjected to downstream QC. **Figure 7** summarizes in a flow chart the time required to complete each major step of the Patch-seq protocol.

## Data Availability

Any raw data supporting the conclusions of this manuscript will be made available by the authors, without undue reservation, to any qualified researcher.

## Author Contributions

MvdH, CB, and JE designed and optimized the protocol with support from FG and GY. MvdH and CB performed lab experiments and analyzed data. CB and FG supervised the project. MvdH wrote the first draft of the manuscript. All authors contributed to manuscript revision and read and approved the submitted and final paper.

## Conflict of Interest Statement

GY is co-founder, member of the Board of Directors, on SAB, equity holder, and paid consultant for Locana. The terms of this arrangement have been reviewed and approved by the University of California, San Diego in accordance with its conflict of interest policies. The remaining authors declare that the research was conducted in the absence of any commercial or financial relationships that could be construed as a potential conflict of interest.
