# Corrigendum: Patch-Seq Protocol to Analyze the Electrophysiology, Morphology and Transcriptome of Whole Single Neurons Derived From Human Pluripotent Stem Cells

**DOI:** 10.3389/fnmol.2019.00150

**Published:** 2019-06-11

**Authors:** Mark van den Hurk, Jennifer A. Erwin, Gene W. Yeo, Fred H. Gage, Cedric Bardy

**Affiliations:** ^1^Laboratory for Human Neurophysiology and Genetics, South Australian Health and Medical Research Institute (SAHMRI) Mind and Brain, Adelaide, SA, Australia; ^2^The Lieber Institute for Brain Development, Baltimore, MD, United States; ^3^Department of Neurology, School of Medicine, Johns Hopkins University, Baltimore, MD, United States; ^4^Department of Cellular and Molecular Medicine, University of California, San Diego, La Jolla, CA, United States; ^5^Department of Physiology, Yong Loo Lin School of Medicine, National University of Singapore, Singapore, Singapore; ^6^Laboratory of Genetics, Salk Institute for Biological Studies, Sanford Consortium for Regenerative Medicine, La Jolla, CA, United States; ^7^Flinders University College of Medicine and Public Health, Adelaide, SA, Australia

**Keywords:** patch-seq, single-cell RNA-seq, induced pluripotent stem cell (iPSC), human neuron transcriptome, neuronal diversity, cellular phenotyping, patch clamping, electrophysiology

There is an error in the Funding statement for GY. GY was supported by grants MH107367 and HD085902 from the National Institutes of Health and the Seed Grant BRFSG-2014-14 from the Brain Research Foundation.

As a result, a correction has been made to the **Funding**:

“This work was supported by the Netherlands Organisation for Scientific Research (NWO) Rubicon Fellowship (019.163LW.032) (to MvdH); the Brain Foundation, the Walker Family, and the Perpetual Impact Philanthropy (grant IPAP2017/0717) (to CB); the G. Harold & Leila Y. Mathers Charitable Foundation, JPB Foundation, and the NIH (Grants MH095741, MH092758, and U01 MH106882) (to FG). GY was supported by MH107367 and HD085902 from the National Institutes of Health and the Seed Grant BRFSG-2014-14 from the Brain Research Foundation.”

Additionally, in the original article, there is an error. The conflict of interest statement is incomplete.

A correction has been made to the **Conflict of interest** statement:

“GY is co-founder, member of the Board of Directors, on SAB, equity holder, and paid consultant for Locana. The terms of this arrangement have been reviewed and approved by the University of California, San Diego in accordance with its conflict of interest policies. The remaining authors declare that the research was conducted in the absence of any commercial or financial relationships that could be construed as a potential conflict of interest.

The authors apologize for these errors and state that they do not change the scientific conclusions of the article in any way. The original article has been updated.

